# An explorative qualitative study of barriers to the use of health and mental health services among migrant female sex workers in Germany and needs for action

**DOI:** 10.3389/fpubh.2024.1464307

**Published:** 2025-01-14

**Authors:** Anastasiia Lotysh, Hürrem Tezcan-Güntekin, Olivia Kalinowski, Gizem Kaya, Franziska Kroehn-Liedtke, Hristiana Mihaylova, Meryam Schouler-Ocak, Wulf Rössler

**Affiliations:** ^1^Psychiatric University Clinic of Charité at St. Hedwig Hospital, Department of Psychiatry and Neurosciences, Berlin, Germany; ^2^Berlin School of Public Health, Berlin, Germany

**Keywords:** migrant female sex workers, barriers, health services use, needs for action, qualitative

## Abstract

**Background:**

Migrant female sex workers (MFSWs) can be exposed to various health risks due to their occupation, including mental and physical health, substance use, and experience of violence. However, they face substantial barriers to accessing healthcare services. The inadequate access to medical care for migrant female sex workers poses a challenge to the German healthcare system.

**Research aims:**

This qualitative study aimed to identify and analyze the barriers to the use of health and mental health services by migrant female sex workers in Berlin and what should be done to improve the access to healthcare and to make it easier to use health and mental health services for migrant female sex workers. The data collected can be used to derive overarching recommendations and strategies for action.

**Methods:**

Semi-structured, guided interviews were conducted with 10 migrant female sex workers in Berlin, Germany. The interviews were audio recorded, and the content of the transcribed interviews was analyzed. A structuring qualitative content analysis, according to Kuckartz, with deductive-inductive category formation was conducted in MAXQDA 2022.

**Results:**

Barriers were analyzed at three levels: patient, provider, and system. The patient level was related to the patient characteristics: social structure variables, health beliefs and attitudes, personal enabling resources, community enabling resources, perceived illness, and personal health practices. The provider level was related to the provider characteristics: skills and attitudes. The system level was related to the system characteristics: the organization of the healthcare system on local and national levels. Moreover, needs for actions were identified, which can be used for deriving recommendations for the improvement of healthcare situation of migrant sex workers living in Berlin.

**Conclusion/discussion:**

Health services and future intervention studies should consider barriers identified in this study to improve the health services utilization and health of sex workers as part of the effort to protect the right of humans to health.

## 1 Introduction

According to the Fondation Scelles report from 2012, sex work involves approximately 40–42 million people worldwide (1). Experts estimate that Germany has approximately 200,000 and 400,000 active sex workers (2). In total, 90% of sex workers are female, and more than half are migrants (3, 4). At the end of 2020, approximately 24,900 sex workers and 2,285 prostitution businesses were registered with the German authorities. Just 20% of all officially registered sex workers in Germany have German citizenship. The three most common foreign nationalities of registered sex workers in Germany were Romanian with 8,800 (35%), Bulgarian with 2,800 (11%), and Hungarian with 1800 (7%) in 2020 (5).

Migrant populations face distinct barriers to accessing healthcare, particularly in the realm of sexual and reproductive health services. For migrant female sex workers, these challenges are compounded by legal, social, and structural factors that hinder their ability to seek timely and appropriate medical care. In Germany, where migrant sex workers represent a substantial portion of the sex work industry, these barriers can be particularly pronounced. These individuals often find themselves in a precarious legal and social status, contributing to their exclusion from essential healthcare services (6). As such, understanding the obstacles faced by this group is crucial for developing targeted interventions to improve their health outcomes.

Existing research highlights how the intersection of migration, sex work, and health creates layers of structural vulnerability, which can leave migrant female sex workers with limited access to services designed to meet their unique needs (7). Factors such as geographic mobility and precarious legal status exacerbate these vulnerabilities, particularly for those who experience frequent relocations or face difficulties obtaining work permits (8). Migrant sex workers, such as other marginalized migrant groups, encounter barriers that include limited health insurance coverage, fear of discrimination, and unfamiliarity with the local healthcare system, making them particularly vulnerable to gaps in health service access (9).

Research has shown that marginalized groups, including queer refugees and migrant families, often navigate health systems from positions of liminality, where their uncertain status compounds their exclusion from services (10, 11). For migrant sex workers, this sense of liminality is further aggravated by the stigma surrounding their work and the cultural differences they face when seeking healthcare (10).

The provision of healthcare services to marginalized populations, particularly migrant female sex workers, presents a significant public health challenge in many countries, including Germany. Despite the numerically large group and its vulnerability, little attention is given to the health status and medical care of sex workers around the world. Women engaged in sex work differ in their level of education, life circumstances, biography, social group origin, or motives for practicing. The forms of practice and areas of activity, which are usually differentiated from one another according to the characteristic of contact with clients, are also heterogeneous (12). Nevertheless, sex workers can be at risk of a whole host of human rights: abuses, including rape, violence, trafficking, extortion, arbitrary arrest, and detention, forced eviction from their homes, harassment, discrimination, exclusion from health services, forced HIV testing, and lack of legal redress (13). Sex workers are exposed to various health risks due to their occupation, including mental and physical health, substance use, and experience of violence (14). The prevalence of mental illnesses such as depression, post-traumatic stress disorder, and suicidality and the prevalence of sexually transmitted infections (STI) and human immunodeficiency virus (HIV) among female sex workers are higher than among the general population (15, 16). Equal access to healthcare is a fundamental human right (17). However, migrant sex workers face substantial barriers in accessing prevention, treatment, and care services, mainly due to stigma, discrimination, and criminalization. Fear of stigmatization, unclear residency status, or lack of health insurance may mean that many sex workers are not reached by regular healthcare services (18–20). These factors lead to the underutilization of the services offered (21) and health inequalities (22). Efforts to address these challenges must focus on culturally sensitive approaches and structural interventions that reduce barriers to care, such as the expansion of migrant health services and legal reforms that protect sex workers from discrimination (23).

While studies have explored healthcare access issues for sex workers in other European countries, the particular experiences of migrant sex workers within the German healthcare system remain understudied. Given the significant migrant population within Germany’s sex work industry, there is an urgent need to better understand the barriers they face and identify strategies to improve service delivery. The lack of knowledge limits the promotion and preventive actions in various levels and contexts. The general objective of the study is to determine the barriers that may restrict migrant female sex workers from using health and mental health services in Berlin and what should be done to improve public health support services for MFSW. Furthermore, the data collected can be used to derive overarching recommendations for action. The perspective of women who are active in the environment of sex work and have a migration background was recorded here firsthand.

## 2 Methods

### 2.1 Theoretical framework

The model of Scheppers et al. (24) of barriers to the use of health services used in this study describes the barriers on three levels: patient, provider, and system level, which provides a nuanced framework suitable for our study’s focus on migrant female sex workers. Multidimensional model of Scheppers et al. (24) is based on Andersen’s behavior model of health services and described all three levels in detail in their comprehensive review exploring the same topic of barriers but on the target group of ethnic minorities, who share similar vulnerabilities with the MFSW. We found this model appropriate for this study.

System level is where legislation or regulation by local or national government impacts comprehensive and effective health service provision, for example, medical paradigm, intake procedure and opening hours, consultancy appointments and waiting time, the length of consultation and treatment, translation, printed materials and other media forms, and referral system.

Service provider level means the configuration or operation of services impacts sex workers’ access to support and services. Provider characteristics are skills, behavior, communication style, style of providing information, client approach, bilingualism, translation, and cultural knowledge.

Patient level means sex worker’s internal beliefs and perceptions of service providers and patient resources impact their access to support and services. Such characteristics are age, gender, ethnicity, living conditions, social class and economic status, education, local language skills, knowledge about physiology, disease, health services, and how to use them (24).

### 2.2 Study population

Inclusion criteria were being migrant women or trans women who are active in the field of sex work in different settings as brothels, studios, clubs, and street prostitution in Berlin. Only individuals over 18 years old who voluntarily participated in the interview were included. Furthermore, participants with Russian, Ukrainian, German, and English language competencies were included. Exclusion criteria were refusal to participate, severe cognitive impairment, highly psychotic decompensation, and acute suicidality.

### 2.3 Sampling strategies

Data collection was conducted from June 2022 till August 2022 within the framework of the “Psychsex” study of Charité Universitätsmedizin Berlin, which investigate mental health of sex workers and was funded by the German Research Association. To better reach the target group, a participatory approach was pursued. Through cooperation with organizations and their member centers (counseling, contact points, and women’s emergency calls), the announcement and recruitment of study participants were taken place directly in places where sex workers receive low-threshold support and counseling, such as Hydra e.V. or Frauentreff Olga. Moreover, informal and community-based networks were contacted. In addition, the recruitment took place directly in the working settings of potential participants (streets, studios, and clubs). Three potential participants were contacted directly on the street, and four studios and three clubs were contacted via E-mail. To ensure voluntary and informed participation, women were approached respectfully and provided with information flyers in their native language. These flyers detailed the study’s purpose, the voluntary nature of participation, and the anonymity and confidentiality of their involvement. Potential participants were encouraged to take the flyer with them and consider participation at their own pace, free from any immediate pressure. Self-reported recruitment strategy was also used when participants found information about the study via websites, information flyers, and social media platforms and registered themselves to participate. In addition, a snowball system was performed, and each participant was informed that we are searching for other participants at the end of the interview (25). However, it is impossible to monitor who joined the research from the snowballing or from self-reported strategy. The interview partners received an expense allowance in the amount of 50 Euros. The decision to offer 50 Euros as compensation was influenced by an understanding of the differing economic contexts faced by participants. For many MFSWs, who may operate in precarious situations, this amount represents a significant incentive. However, we also recognize that perceptions of value can vary widely among participants.

We sought to ensure that the compensation was equitable and reflective of the time and effort required to participate, while also being mindful of the economic realities many MFSWs face. By providing compensation, we aimed to mitigate potential barriers to participation and encourage a broader representation of voices within the study.

In this study, special attention was given to ensuring anonymity and building trust with participants, particularly given the vulnerable nature of the MFSW population. These considerations were crucial in encouraging open participation and mitigating fears around legal repercussions or breaches of confidentiality.

To protect participants’ identities, no identifiable information was collected or recorded, such as names, addresses, or contact details. Pseudonyms were used throughout the study, and any potentially identifying contextual details were removed or anonymized in the final analysis. Data collection and storage followed strict confidentiality protocols, adhering to local privacy regulations and ethical guidelines. Participants were informed that they could withdraw from the study at any time without providing a reason, and their data would not be used in the analysis, further reducing any perceived risks of participation.

Trust was established by working closely with intermediary organizations that had existing, positive relationships with the MFSW community. These organizations, including support groups, health service providers, and NGOs, played a key role in facilitating recruitment and engagement by vouching for the research team’s credibility and explaining the purpose of the study in a way that aligned with participants’ needs and concerns. The endorsement from these trusted entities helped alleviate fears of exploitation or exposure. Participants could choose the interview setting where they feel safer; partner organizations have provided a familiar and supportive environment for recruitment and conducting of interviews, such as meeting in spaces where MFSWs felt comfortable and trusted. This helped reduce the anxiety some participants might have felt about speaking with outsiders.

By ensuring anonymity and leveraging the trust established by intermediary organizations, we were able to create a safer, more secure environment that encouraged MFSWs to share their experiences more openly.

### 2.4 Data collection

Data were collected using semi-structured guided interviews according to Groeben and Scheele (26). When possible, the interviews were conducted in the native language: Russian or English, with a duration of approximately 20–45 min. The interviews were audio recorded. Ten interviews were conducted, two of which took place online and eight face-to-face in the project room of “Psychsex” study in Charité Campus Mitte. When from research team point of view the field of knowledge has been saturated, the data collection was closed.

The interview guide was developed based on the state of research and the model of Scheppers et al. (24) already used to describe the barriers to the use of health services. The SPSS scheme of Helfferich (27) and his principle: “As open as possible, as structuring as necessary” was used for the development of the interview guide. An interview guide provides an open-ended introductory question for each topic area and corresponding specific follow-up questions.

We aimed, for participants, to provide genuine accounts of their experiences, and the presence of compensation might have led some individuals to tailor their responses to align with what they perceived as the expectations or goals of the study. Participants may have felt compelled to share experiences that they believed would be more favorable or acceptable in the context of the research, rather than expressing their authentic feelings or situations.

To address this concern, we implemented several strategies during the data collection process. Interviewers were trained to maintain a neutral stance and encourage participants to share their true experiences without fear of judgment. They reinforced that there were no “right” or “wrong” answers and that all experiences were valid. We emphasized the anonymity of participants, reassuring them that their responses would not be linked back to their identities, which aimed to create a safe space for open dialogue. We used open-ended questions, which allowed participants to express themselves freely, thereby minimizing the risk of skewed responses influenced by the compensation.

### 2.5 Data analysis

The interview audio files were transcribed in MAXQDA 2022. Transcription rules for computer-assisted analysis, according to Dresing, were used (28). Eight of ten interviews were in English. Direct quotes from one interview in Russian and one in German that are conducive to answering the research question were translated into English. A structuring qualitative content analysis, according to Kuckartz, with deductive-inductive category formation in MAXQDA 2022 was conducted (29).

The analysis, according to Kuckartz, was conducted step by step. The coding process began with an initial round of open coding, where interview transcripts were systematically reviewed, and key themes and concepts were identified. The authors made initiating text work and case summary. Then before coding, main categories were deductively derived, according to the barriers model used by Scheppers et al. (24) and the interview guide. After that, all the materials were coded according to main categories, which were supplemented by subcategories that were inductively created on the material (29, 30). In the end, every interview was recoded with the whole system of main and subcategories. Further material analysis was performed in the form of tables (29). To ensure a robust coding framework, codebooks were developed, detailing the definitions and inclusion criteria for each category. This helped in standardizing the coding process and ensuring that the same themes were applied consistently across all transcripts.

To enhance the reliability of the analysis, the study incorporated inter-coder reliability checks. Two independent coders were involved in the coding process. After the initial round of coding, a subset of transcripts was double-coded by both researchers. Any discrepancies were discussed and resolved through consensus meetings, which led to further refinement of the coding scheme.

## 3 Results

The information gained from the interviews could be organized into four main categories: barriers on the system, provider, and patient level, and needs for actions (see Table 1).

**Table 1:** Summary of main and subcategories.

Main category: barriers on the system level	Subcategories: A complex health system Long waiting times Uncovered services Lack of available information
Main category: barriers on the provider level	Subcategories: Stigma Phoning Language barrier Misdiagnosing Do not take seriously/do not listen to Gaslighting Unprofessional behavior Cultural differences Availability Market orientation
Main category: barriers on the patient level	Subcategories: Lack of health insurance Lack of system knowledge Language knowledge Self-stigma Being a freelancer Mental health issues Accessibility nearby High medical costs
Main category: needs for action	Subcategories: Awareness raising and sensitizing the doctors Centralized website Ensuring anonymity Mental health support Shorter waiting times More available languages Legislation and support with legislation More specialized services Better advertising Availability

### 3.1 Sample characteristics

The age varies from 21 to 46 years. Three participants are registered as a sex worker and have prostitution passport. At the moment of the interview, just one of the participants did not have German health insurance (B5), and three were not insured earlier (B7, B8, B4). All who are insured have statutory health insurance. Some sex workers are freelancers, some work in an institution such as clubs or studios, and some are street-based. Two of the sex workers are trans women and homeless (B7 and B8). It could be assumed that one of the participants was under the influence of drugs during the interview (B7) (see Table 2).

**Table 2:** Study population characteristics.

ID	Country of origin	Registration as SW	Health insurance status
B1	Italy	No	yes
B2	USA	Yes	yes
B3	Australia	No	yes
B4	England	No	yes
B5	Australia	No	no
B6	Iceland	Yes	yes
B7	Lebanon	No	yes
B8	Czech Republic	No	yes
B9	Russian Federation	Yes	yes
B10	Italy	No	yes

*SW—Sex worker.

### 3.2 Barriers on the system level

On the system level are barriers where legislation or regulation by local or national government impacts comprehensive and effective health service provision. This main category was organized into four subcategories:

#### 3.2.1 Complex healthcare system

*“like at one point I felt like it was like a part-time job, just trying to organize and schedule and go to appointments for health stuff. So, yeah, it's quite difficult. But the care that I have received has been quite good in some areas when I finally get to it.” (B4, Pos. 26)*

The participants find the system so complex that one compares the organizational process to a part-time job and simultaneously describes the care as good but hard to access. Migrant sex workers spend much time and energy trying to access the care. Some participants also mentioned that they would go more often if access would be easier.

#### 3.2.2 Long waiting times

One of the central issues for MFSW by searching for medical help is long waiting times, especially for psychotherapy and gynecologists:

“…gynecologist, which I probably should have. Yeah. But, I tried to […] and the waiting list for them were like six months. So I just don't go to gynecologist.” (B3, Pos. 40)

All participants mentioned that it would be better to have a chance of getting the treatment faster because sometimes they have some acute issues because of specificity of their job and cannot access the system urgently:

*“And the other problem is like, when you try to get an appointment, it's always like after months. I can understand that because Berlin is big. We are a lot of people, so yeah. But sometimes it's just, it would be better like to get a closer appointment, because if you have some problem then it's complicated.” (B10, Pos. 12)*

#### 3.2.3 Uncovered services

Some participants mentioned services that are not covered by health insurance. However, they would like to have the possibility to get it for free, for example, STD and HIV testing by a gynecologist or by “Hausarzt” General Practitioner (GP), acupuncture and dental services, such as teeth cleaning and caries treatment:

*“I have one where I could go if I had an emergency, but I don't like her. And she makes it paid for STI tests and stuff. She told my friend who's not a sex worker. It would only be free if she was trying to get pregnant or pregnant. Yeah. So I don't like her.” (B3, Pos. 40)*

#### 3.2.4 Lack of available information

One of the problems in the system is that information about existing help services is not disseminated in places where the target audience is located, for example, in working settings. The prevention dilemma here is that those who most need it cannot reach the offer:

*“I would also say, I've never seen any information in the clubs or anything like maybe just one small leaflet of… these are good resources, these are trusted.” (B4, Pos. 38)*

### 3.3 Barriers on the provider level

The configuration or operation of services and perceptions or behavior of service providers impact sex worker’s access, and the use of support and health services means a provider level. The second main category can be organized into ten subcategories:

#### 3.3.1 Stigma

Stigma is a common phenomenon for migrant sex workers within the healthcare system. MFSW can experience multiple forms of stigma regarding sex work, their origin, mental illnesses, or STI. However, in most cases, they experience occupational stigma. Stigma can cause different scenarios: Sex workers do not trust the doctors, do not want to be open and honest, hide some information that could be relevant and have an impact on the treatment process, use fewer health services, and avoid visiting doctors. The participants experienced some situations in which psychotherapists and some specialists such as internists or gynecologists were stigmatizing by the appointment:

*“But my psychotherapist, she knows that I'm a sex worker, so she knows that I do stripping mm-hmm, but she doesn't know that I also do full service. Because I have noticed that sometimes she said some things that were a bit judgmental towards prostitute. So, and stripping is more accepted, so I'm like, okay, I just say that part.” (B1, Pos. 33)*

#### 3.3.2 Phoning

One of the participants said that the phoning and speaking are frustrating, and the specific phone hours is difficult to reach some clinics, make an appointment, or get the information. Most prefer to use Internet and some websites where they can make a doctor’s appointment or search for a doctor:

*“I feel like phoning is a big barrier to actually getting some of the information that you need to then move on […] I found the specificity of phone hours was difficult for me to really get a hold of someone and then the actual act of doing it and speaking was frustrating.” (B5, Pos. 34)*

#### 3.3.3 Language

Language as a barrier on the provider level means the language knowledge of health services providers, prolonged waiting lists for English-speaking doctors, difficulties in communication during the medical visit, especially by psychotherapy, and refusing to provide health services because the patient does not speak the local language:

*“…but the issue is always the language. I scare when I go to hospital or to the doctor. It's happened to me in (name) hospital in (name of the street). The doctor, she refused to speak in English. So, she asked me to leave. She said I cannot help you. I cannot make for you anything. So, I get very angry and I was shocking. It's possible to bring anyone to translate between us. But you look like just wanna kick me out. You don't want even to try. She say, it's not my, it's not her problem. And she asked me to leave and I was getting very mad from her because she, she put me in very bad situation. After waiting I think I remember maybe for half an hour or more, she just asked me to leave, because I do not speak German and I told her that it's impossible for me. I could English and, and she refused.” (B7, Pos. 34)*

#### 3.3.4 Misdiagnosing

One of the participants was misdiagnosed with one fatal disease by a gynecologist and was sent to the hospital. Important to mention that this case of misdiagnosing is not related to sex work stigma and could happen to other patients too. She described being afraid, scared, and strongly believed in the diagnosis:

*“…weird actually, felt weird because like, I mean, with this like (diagnosis), it was really bad. And also like when I went to the hospital like they treat me a bit like crazy because I was sure about it because, you know, she told me that and I was like a bit scared. But they were like, no, there's nothing at all. Like, I don't believe them. I was like, okay. But it is my doctor, so, you know?” (B10, Pos. 14)*

Such situations can cause mistrust to the health system, the doctors, and provider’s expert knowledge and, as a result, insufficient use of health services.

#### 3.3.5 Do not take seriously/do not listen to

Some participants talked about experiences with specialists and GPs, where they did not feel being taken seriously or that their complaints were not taken seriously. They described not getting personalized help that they felt not to be heard:

*“I don't feel like I get really like in depth or personalized help.” (B 2, Pos. 22)*

*“no, I tried a few, but I I'm still in a search of this, because like, most of them, like they're, they don't take it like personally, you know, like it's just, you are just a number, so yeah, it's a bit difficult.” (B10, Pos. 8)*

#### 3.3.6 Gaslighting

Almost every participant reported about gaslighting experience within the healthcare system. The symptoms were arbitrarily dismissed as insignificant. These experiences can be traumatized, and one participant said that she sometimes neglected health issues for this reason. Gaslighting generated different negative feelings and reactions by participants: crying during the appointment, nightmares, discomfort, anxiety about the system, feeling of being infantilized, dismissed, frustration, being scared, and being angry:

*“I just felt that they, uh, they just didn't believe me. Mm-hmm, or that they didn't take seriously my pain or my diseases. So you are, yeah, it just made me feel a bit like, like a kid. You go to the doctor and they tell you ‘no, well, your pain is not so bad, no, you're not really sick.’ So, yeah, I just felt infantilized.” (B1, Pos. 18)*

*“I have some strong issues with pain, which I think might be directly related to the sex work and stripping for 14 years. Um, and I feel like when I've had specialist appointments, they do not particularly listen to my experience: ‘you are quite young, like no you shouldn't be getting this pain’, not realizing that actually it's like a very intensely, wearing job on certain things issues were very ridiculous.” (B4, Pos. 26)*

#### 3.3.7 Unprofessional behavior

Some participants reported different situations when doctors’ behavior caused discomfort and bad feelings during the appointment:

*“I had one experience, uh, one appointment with a neurologist who there was an extremely uncomfortable appointment who asked me to get undressed from the pants for the vape. Whatever is that, what's the word… checkup thing. And then left me, sat in my pants for like 15 minutes while he was typing away. And yeah, that was just super uncomfortable.” (B4, Pos. 26)*

*“Then I've been to another one and he did the like pap test without saying me this. He told me only after, like he goes, he was like: ‘maybe you are afraid of it or so’. Okay, thank you. Maybe I want to know that.” (10, Pos. 12)*

In the second situation, the doctor performed a gynecological test without warning the patient, which is a paternalistic approach. Furthermore, the doctor seems to think he knows what would be better for a patient and deprives her of choice and decision-making.

#### 3.3.8 Cultural differences

One of the barriers which can lead to difficulties, misunderstanding, miscommunication, and discomfort by MFSW during medical visits is cultural differences such as body language, gestures, mindsets, communication, manners, and norms:

*“So I think it was, yeah, didn't especially coming from the UK where I think the services are fantastic. Not that the services are not, I think the medical services in Germany are great, but I'm used to very overly friendly staffed. So, I think there's a bit of a cultural difference as well. That was quite difficult at first.” (B4, Pos. 26)*

*“German doctors, not all of them, but a lot of them are very cold” (B1, Pos. 44)*

#### 3.3.9 Availability

The availability of doctors was characterized by a high patient load, limited capacity, and a lack of free appointments. This issue is particularly acute among sex work-positive doctors. Access to general practitioners (GPs) is especially significant for sex workers, as they often serve as the primary point of contact for essential health checkups related to their profession, such as STD testing. However, GPs are frequently overwhelmed, further complicating access to necessary care:

*“But for example, also my like Hausarzt is very busy and it's not very easy to go if I just have something that I want to check up on.” (B5, Pos. 8)*

Overwhelming of doctors in Berlin causes MFSW difficulties because they mostly visit the doctors with a recommendation from the community or a trusted resource, and this number of doctors is limited.

#### 3.3.10 Market orientation

Some participants reported situations when doctors tried to sell some services aggressively, which was not the goal of the visit and out of the patient’s request. Another side is when doctors are not interested in a treatment result, and patients continue to visit and pay them without a result:

*"Now somehow it's also more on this Market. Well, I think it's even more the point that doctors are selling more […] he told me that I don't need to take your blood five times, let you come in for a biopsy or two and that means he wants to cut out my organs. He says I'll get more. He says I'll get more. So, he said […] he said straight away that I wouldn't make any money with you if you come here for the paperwork" (B9, Pos. 44).*

### 3.4 Barriers on the patient level

The patient level is when sex worker’s internal beliefs, perceptions, and financial and social status impact their utilization of health services. This category was organized into eight subcategories:

#### 3.4.1 Lack of health insurance

Lack of health insurance causes insufficient use and access to healthcare services. MFSW reported situations when they could not receive medical help because they were or are uninsured:

*"… when I didn't have health insurance. And I was very sick and I had a lot of pain and they couldn't help me." (B8, pos. 36)*

*“So that was really bullshit […] he couldn't really offer me help. He kind of said that I should go to like, go back to (country of origin) and that I should leave sex work. And that like, because I, because of the insurance that I had, that he couldn't help me.” (B5, Pos. 14)*

In the second case, the participant has insurance, but it is a touristic one with limited access to care. The doctor not just refused to help but has made some inappropriate instructions. The doctors need to be sensitized to work with people without health insurance or be trained with other types of insurance, such as touristic. The situation of being uninsured causes a lot of stress and insufficient use of health services:

*“Because I was really, really anxious at some. I was really struggling with physical health and mental health at some point, and realized I was in quite a precarious situation if something maybe did happen, I did not have any healthcare. And then I knew maybe I could use sometimes the XXX card (touristic insurance), but I was also, I didn't want to end up in a situation that I had a huge hospital bill or something that I couldn't pay.” (B4, Pos. 18)*

#### 3.4.2 Self-stigma

Almost all participants reported sex work self-stigma, which is manifested by the fear of stigma, which could come from doctors, the fear of being judged, unknown reactions, shame, and embarrassment. Sex workers with self-stigma are less likely to seek healthcare, be honest with doctors, and tell the truth. They aspect from the beginning, the judgments toward their profession from the healthcare providers and prefer to hide it or lie. This expectation is justified by the fact that they personally or in their community have had negative experiences with doctors. Some sex workers stigmatized themselves also because of some health issues related to the job:

*“…an appointment I went to recently, it was an issue. And it was actually more of an issue with me being a sex worker. It's quite embarrassing. I had XXX (illness) and for most people I feel like it's, oh, maybe that's not such a big issue, but for me it could possibly mean that I can't work for a while.” (B4, Pos. 30)*

They also have a fear of not getting the same level of treatment or being misdiagnosed because of stigmatization of sex work:

*“I'm fearful for it that there's judgments or that, that they don't give the same level of care in a way that it would affect, whether they realize it or not as well. I think sometimes it can be subconscious bias also. Um, or I'm not fully honest, like it was the case where I say I'm a yoga teacher. Which is then there's, it's not the correct information ultimately that might be needed. Um, yeah. It's, it's quite a scary thing to go.” (B4, Pos. 34)*

*“…so I didn't say anything also because yeah, I'm just scared that if I say something then stigma comes or they're gonna tell me that, oh, it's my job, that makes me sick.” (B1, Pos. 33)*

At the same time, sex workers want to be honest with the doctors and understand the importance of telling the truth, but it is difficult to be open:

*“It is a bit weird because it, it would be better to tell the truth, you know? It would be clear for everything that can happen, you know? But it's a bit tricky.” (B10, Pos. 30)*

#### 3.4.3 Lack of system knowledge

The next hindrance to medical care is a lack of knowledge of how the system works, whom to contact first, and where to get a prescription. MFSWs describe being confused, unsure, and afraid of making appointments because they do not know whom to go to first. They compare the system with the healthcare systems of the countries where they have lived before and describe it as difficult to understand, especially during the corona pandemic. Moreover, migrant sex workers should consider many aspects (sex work positiveness, language, and location) by looking for doctors, which weakens access to healthcare. So, they need to invest much energy to understand the system and find the right doctor:

*“So after that, I was like, not really sure what, what to do. It's a bit confusing to try to figure out where to go for help and who would be like, you know, supportive of my work and like queer community and stuff like that. So, I, you know, I wanted to put myself in a safe place. So unfortunately I haven't found anybody yet because it's, maybe I put it off because it was like a little bit complicated to try to figure out where to find help and stuff.” (B2, Pos. 8)*

*“So, it was also trying to understand the system […] and it, I felt at times it's just so much energy to try get anything sorted that I gave up at times, and then I'll leave it a few months and then I have really bad pain again. And then I go back again and it's just kind of back and forth constantly.” (B4, Pos. 26)*

#### 3.4.4 Being a freelancer

There are some difficulties caused by being a freelancer. The first one is that freelance sex workers have no paid sick leave. At times being ill, they lose their money, and consequently, they try to avoid sick leaves and search for a faster solution, such as medicine, for example:

*“But if you are a sex worker, sex workers are freelancers. You cannot stay at home for two weeks drinking tea, because you are not paid. You don't have a sick, any sick leave.” (B1, Pos. 12)*

The second point is that sex workers can have work problems with the club’s due to a work cancelation caused by a doctor’s appointment or sick leave:

*“… if you need a doctor's note to take off work, I've had an issue before that, because we're, self-employed and that often, like there was one day I had to cancel a shift for work, which technically as self-employed it's it then becomes a bit of an issue with the worker status kind of thing.” (B4, Pos. 42)*

#### 3.4.5 Mental health issues

Some participants described mental health issues as a barrier to the use of health services. Participant describes her feelings in general about the visiting doctors and talking about complaints:

*“…because it's difficult. It's difficult to go to a new place and like childhood traumas and explain to them why I'm there. Why I have XXX to like convince them. That like, this is, this is how I feel. I always get, okay, why is there really thing in your life? And I like have to feel like I have traumas that I have to do with this, this and this, and that's incredibly difficult. And I have to do it again and again and again. It’s that, yeah. So I find it difficult. I find it like afterward I was really uncomfortable because I'm not necessarily ready to do to talk about these things in that way or that order, or, you know.” (B6, Pos. 20)*

#### 3.4.6 Accessibility nearby

The logistical question can cause a difficulty, especially when MFSWs move around the city or country a lot. They found the doctors and then, after moving, should search for another one because the previous one is far away to visit him/her for something acute. Another point is that some specialists are hard to find in some districts, or recommended and popular in community doctors are often far away:

*“Another thing is just that, like all the good ones are just on the other side of the of the city.” (B10, Pos. 20)*

#### 3.4.7 Language knowledge

Migrant sex workers do not have enough German knowledge to explain their health situation by doctor’s appointment or visit the therapy they need. They struggle with difficulties such as finding an English-speaking doctor or finding information in English or their native language, being embarrassed to speak bad German:

*“The language, the language barriers also were really difficult because just trying to find accessible English health service and information.” (B4, Pos. 22)*

#### 3.4.8 High medical costs

Freelance sex workers should pay by themselves for health insurance, or if they are undocumented or want to have some extra services, they pay privately, often for therapy, for example. Some participants reported that it is a big amount of money. Due to the reason that they earn differently every month, and especially during the lockdown, it can be difficult to pay privately for some health services:

*“…if you don't earn that much money, then that's a lot to pay every month.” (B2, Pos. 24)*

*“…and you know, budget. Cause like I'm not gonna be able to pay private during like the lockdowns and around that time.” (B6, Pos. 16)*

### 3.5 Needs for action

This chapter introduces wishes and thoughts of MFSW about the changes which should be done to make use of health and mental health services more accessible.

#### 3.5.1 Awareness raising and sensitizing the doctors

The first point is the importance for sex workers not to be judged in the healthcare system and to feel safe and comfortable during the appointment. They wish that doctors would have no stereotypes and stigmatization regarding the profession and not transfer their prejudices on sex workers during the medical visit that doctors stay unbiased:

*“…is when a GP or a doctor or someone or a psychiatrist isn't projecting or assuming stereotypes of what they have, which can be a barrier to what I'm actually saying or for, to them to hear what I'm saying […] I think like the most important thing for accessing care, I guess, is that like, okay, you can hear what I'm saying, you're listening to me and you don't think, you know, better than what I'm saying, or you're not trying to find out something that I'm not telling you.” (B5, Pos. 36)*

Antidiscrimination and antistigmatization training and awareness-raising courses about sex work, trans life, and people without health insurance for medical professionals and health and social assistance administrators are needed nationwide.

#### 3.5.2 Centralized website

One of the main resources for MFSW is the Internet. For this reason, half of the participants said that medical care would be more accessible in Germany with one well-known centralized website available in different languages, where people could search for information and doctors and filter them by district, languages, and sex work positivity. One of the available functions of this website should be the ability to book appointments online:

*“…that would make it much more accessible or much, much easier. Like, you know, a very good website design, very clear information, information available in different languages. Also a very clear portal or something also not necessarily having to phone […] So it's good to be able to access as much information as possible, like kind of online gather things and then be able to get in touch with someone or arrange a phone call or something.” (B5, Pos. 34)*

#### 3.5.3 Ensuring anonymity

The opportunity to stay anonymous by getting medical care is important, especially for undocumented or not registered sex workers. Anonymity could help to avoid the stress caused by fear of being arrested or deported:

*“…people often don't feel safe to do so, but like I said, if, if they're given a place where they could be anonymous, where they don't have to worry about being registered or being illegal or anything like that, where they just know they can get help and someone's gonna listen to them. I think that that would make all the difference in the world, at least for me and others, you know?” (B6, Pos. 40)*

#### 3.5.4 Mental health support

MFSWs face various barriers to getting mental health support, which suggests their need is uncovered. Some participants reported that sex work influences their minds. If sex workers could get mental health support in time, it would be possible to avoid some consequences as burnout or substance abuse:

*“I mean also, yeah, like the therapy, like for mental health would be nice to get it, like if you're a sex worker, because it brings a lot to your mind and would be nice. I mean, at least like one or two appointments, like in a month that you can just get to go and talk to someone”(B10, Pos. 48)*

*“…we have a good team we talk to each other in our studio with my colleagues but sometimes you want to talk about clients or about this stuff with someone who will understand you even medically, to avoid consequences like burnout and all the other things. Or don't go into alcohol or whatever.” (B9, Pos. 36)*

#### 3.5.5 Shorter waiting times

The waiting times should be improved for all doctors, especially psychotherapists and gynecologists. It would be helpful if MFSW could get quick access or the information they need in urgent cases because they have direct contact with blood and other body substances at work and also because of the influence of work on their minds.

#### 3.5.6 More available languages

MFSWs wish more available information in English or other foreign languages and cultural/language mediators in the health facilities. Another aspect regarding the language is that doctors who have English language knowledge do not refuse English-speaking patients and agree to speak in English with their patients:

*“First, it would be really great if it were more, a little bit more international in the sense of like having like language barriers a little bit broken with that, I know that a lot of them speak English, but there are some that will straight up just not speak English” (B6, Pos. 36)*

#### 3.5.7 Legislation and support with legislation

Undocumented or who was undocumented MFSW would like to receive support in the legislation process, making the correct paperwork and health insurance. When they arrive and do not know how the German system works or a local language, it takes a long time and causes much stress to make everything correctly. Moreover, MFSWs want sex work regulations that protect them from discrimination by official authorities:

*“I think the more the laws and legislation and things protect as well, the more comfortable I feel doing that” (B4, Pos. 40)*

The regulations of sex work should be developed in cooperation with sex work groups.

#### 3.5.8 More specialized services

MFSWs wish more support and more available specialized services, not just STI and HIV testing, but also mental health support, and more medical support for undocumented sex workers for free and anonym. Participants described their situation as vulnerable and at a higher risk of being infected because they work in direct contact with people, with blood and other substances. For this reason, it is important to have an accessible medical center in one place which is well-known and advertised within the community, which include regular medical checkups, blood testing, STI testing, gynecologist, and urologist for information about specific work moments. Moreover, there should be a possibility to receive medical support anonymously in the case of violence because the one place where they could go is emergency or police, but if a sex worker is undocumented is difficult:

*“So you offer for the people who have a normal job, that there is yearly checkup for full checkup. Cause if you're normal staffing somewhere, you have, you get your health checkup, medical every year. I hope that also be include us that we also have every, every six months maybe. Full checkup for all, all kind of health, healthcare […] we should to follow it, like, testing, blood testing, everything to can be sure that we're healthy. Because we're in very sensitive situation. We are in contact with others in blood, in mouth, in everything […] So I hope that we are all having the same, same level of health care […] the most important thing that there is a way to can avoid the bad, bad, bad situation. So one of this way is to, can have this kind of organization who follow up with us every time to can be in the safe side” (B7, Pos. 48,50,52)*

#### 3.5.9 Better advertising

There should be all information when sex worker register, said one of the participants, but the dilemma is that not everybody is registered (B2). So, the information should be available in the workplaces where sex workers spend most of their time. In addition, on the Internet and social media platforms:

*“…but then that needs to be very well advertised within the community, which can be quite a hard thing cause it's quite a closed group sometimes. Um, I think things like Instagram from the group or these Facebook groups, like the, the private Facebook group for the sex workers […] As a preemptive thing, sometimes as well, before you need the care, it would be nice to know that it's available.” (B4, Pos. 38)*

#### 3.5.10 Availability

There should not only the information be available at sex worker venues but also health services, otherwise homeless or undocumented, or drug-addicted or sex workers who have no mobile phones or access to the Internet cannot access all health services they need. The concept of regularly available, free, and anonym health checkups and mental health support directly in the working settings could be helpful for some MFSW too:

*“…like in terms of like marginalized people and people who work on the street, like the support needs to be there. And not hidden behind some facility or in a doctor's clinic on the other side of town, you know, like these people work on the same corner every day and go home or not, if they're homeless and they don't have the resources to even research. Like someone needs to go there and offer it to them because otherwise it just doesn't happen.” (B3, Pos. 50)*

## 4 Discussion

Some categories are not explicitly categorized and can be interpreted differently. One example would be «availability». It could be interpreted as a lack of medical staff as a problem at the system level or as a very popular within the community sex work positive doctor, who is very hard to access. In the second case is a barrier at the provider level. Here, the question arises if this barrier should be presented under barriers at the system or provider level. This question is one of some examples that can be given. The fact that some placements do seem arbitrary does not affect the quality of the results. The arbitrary placement does not change the content of what is stated. However, the reader should be warned and interpret the placements with some reservation.

Our explorative study provides first insights into the barriers and needs regarding migrant sex worker’s access to healthcare from the perspective of migrant female sex workers in Germany. The findings overlap with prior studies and international literature on sex workers and healthcare access but also present unique insights specific to the German context. Global Network of Sex Work Projects says that only services available for sex workers are HIV and STI prevention (31). All participants of this study confirmed this thesis that the only available specifically for sex workers service is HIV and STI checkups, but their needs include also mental health support, sexual and reproductive health consultations, general health, and dental care. Nevertheless, our findings overlap with the finding of German Aids Organization and qualitative study from Bochum that facilities of the public health service play an important role in the sexual health of sex workers, in particular through the free and anonymous HIV/STI screening programs in accordance with § 19 of the Infection Protection Act (19, 32).

There are similarities between the findings of this study with TAMPEP and international literature where stigma was detected as the main barrier to health services among sex workers in general. In addition, that many migrant sex workers avoid public services, mainly hospitals, because they potentially risk detection or deportation if they have not a legal work status (33–37). Stigma, self-stigma, and lack of documents were also detected as main hindrances to health services by MFSW in this research.

Barriers such as language difficulties, lack of culturally sensitive services, and concerns about legal status also resonate with global observations on migrant health (9). The compounded nature of these challenges is particularly evident in MFSW’s hesitance to use public health services, mirroring the structural vulnerabilities discussed by Carruth et al. (7). The difficulty in navigating complex healthcare systems without adequate cultural or linguistic support has been identified across various migrant populations and is reflected in the findings of this study.

Most of the barriers to accessing mental health services by sex workers, which were identified by Macioti et al. (38) in Germany, and barriers to public health services in EU by TAMPEP (39) overlap with the findings of this study: Language barriers were seen as a problem for accessing services by most migrants, need to educate and sensibilize the doctors to the topic of sex work, lack of available places for psychotherapy, high cost of health insurance, lack of options for accessing services anonymously, ID-ing and the practice of recording the full range of a person’s data to a central database, meaning once a person is registered, information on their status is available to all social institutions, fear of deportation, lack of protection from deportation; stigmatizing attitudes and discriminatory treatment toward female, and transgender (migrant) sex workers by public health services; lack of trust in public health institutions due to sex worker’s experiences of exclusion and stigmatization; language barriers and lack of services that are respectful of one’s culture (including information material in several languages, multicultural staff at public health services, and cultural mediators); dependence upon third parties (pimps, brothel owners); lack of access to information on public health services, the structure of the national health system, and service providers (inclusive services run by NGOs); and lack of knowledge of one’s own rights, and resources for public and NGO health services (38, 39).

Further overlapped barriers with international literature were found: self-stigma; difficulties in obtaining a permit of residence and work; overload system (availability); long waiting times (34, 36, 40–44).

One barrier which was reported by TAMPEP (39) and not found in this research is isolation: lack of integration with the local society and sometimes restriction of free movement. The possible reason for this could be that the world has digitalized and through the Internet and social media groups, especially in Berlin, it is now easier for sex workers to develop networks and stay in exchange, and in Berlin, there is no restriction of free movement at the legislative level.

Moreover, in this study were identified new findings that were not found in the literature. Following barriers hinder MFSW access to the healthcare: complexity of the healthcare system, uncovered services by the health insurance, unpractical method for making appointments (phoning), misdiagnosing, when the provider do not take the patient seriously and do not listen to, gaslighting, doctor’s unprofessional behavior, market orientation of providers, being a freelancer, mental health issues, accessibility of services nearby, because of moving to the new places.

Some of the barriers can also be applied to a general population. For example, many patients, independent of their social status, age, and origin, face the barrier of long waiting times. Nevertheless, MFSW reported that in urgent cases, because of direct contact with blood and other body substances or influence of job on their mental state, they sometimes need faster access to doctors. The barriers often lead to the insufficient use of health services. MFSWs do not trust to doctors and have fear and anxiety by visiting of medical institutions. As coping strategies, they avoid seeing doctors, search for some alternatives, such as natural treatments or medicine, search for information on the Internet, or use peer support within the community. The information, experience, recommendation exchange, and support within the community play the prominent role by using the healthcare system.

The findings from this study underscore the need for a multifaceted approach to improve healthcare access for MFSW. Drawing on WHO guidelines (45), healthcare systems must prioritize the principles of stigma reduction, non-discrimination, and cultural sensitivity. The WHO guidelines on HIV/STI prevention and treatment for sex workers 2012 state that all health services, including primary healthcare, should be made “available, accessible, and acceptable to sex workers based on the principles of avoidance of stigma, non-discrimination, and the right to health” (45). Therefore, integrating recommendations from Bouaddi et al. (6) and Carruth et al. (7) and firsthand information from this study, targeted interventions should include the following: Culturally Sensitive Training, which should focus on understanding the unique vulnerabilities of MFSW and ensuring that care is respectful, empathetic, and non-judgmental. Digital Health Expansion: Anonymous, multilingual digital platforms should be developed to provide mental health support, health education, and access to telemedicine. Policy Advocacy: Structural reforms, such as simplifying legal documentation processes and increasing funding for public health services, are necessary to address systemic inequities (6, 7).

Peer-to-peer networks, as discussed in Groeninck et al. (11), also hold promise for improving healthcare access. Participants in our study described relying heavily on community support for information, guidance, and emotional aid. These networks, while informal, act as critical lifelines, compensating for the inadequacies of formal healthcare structures. Expanding and formalizing peer-led initiatives could enhance their impact, aligning with global recommendations for leveraging community resources to address health inequities.

The Robert Koch Institute (RKI), a German federal government agency and research institute, research on Culturally sensitive HIV/STI prevention among migrant sex workers concluded that the free delivery of safe sex material is not enough to prevent STI’s/HIV, and constant sexual health education from an gender perspective is needed (46). The findings in this study showed that not only sexual health education is needed but the providing of information about existing services in working places in different languages or providing of health services in the working settings anonymously, for free and with a regular follow-up. Many studies concluded that interventions for preventing STIs among female sex street workers and sex workers are strongly needed (46–48). However, the results of this study showed that mental health support is also highly needed within the community. The participants mentioned that sex work influences mental health status and that mental health services could prevent some consequences, such as burnout and substance abuse.

There are some overlaps with the finding from previous studies about sex workers in other countries. Nevertheless, the current explorative study offers insights by focusing specifically on migrant sex workers in Germany, a population that has received limited attention in prior research. Notably, this study provides firsthand data gathered directly from migrant sex workers, offering a rare and invaluable perspective on their specific challenges and experiences in navigating healthcare systems in Germany.

Unlike previous research, which may have addressed general barriers faced by sex workers, this study highlights the compounded difficulties encountered by migrants, including language barriers, legal status, and cultural stigmatization. To our knowledge, no previous study has systematically collected such detailed, primary data on this particular population in Berlin. By doing so, this research fills a critical gap in the literature and offers fresh insights into how healthcare services can be made more accessible and responsive to the needs of migrant sex workers. The firsthand information of this qualitative research goes in depth in the needs, experiences, and wishes of MFSW, which allows to improve existing services and create new support services that are needed, such as anonymous, free, well-advertised in the community and easy-to-access mental health support, STI and HIV checkups, and health consultations directly in working settings, available in different languages or with a cultural/ language mediator. Further research is needed on the types of interventions that work best for MFSW: developing of centralized website, information campaigns, walking clinics in the working settings.

### 4.1 Limitations

The qualitative method and semi-structured interviews chosen for this study provided in-depth information and personal experiences and stories of participants, allowing for an in-depth analysis of the study’s topic. However, MFSW is a very heterogeneous group. To address this problem, different recruitment strategies were applied and different women, as well as trans women, working on the street, clubs, studios, hotels, escort, and massage salons have participated. Nevertheless, the recruitment strategy, which primarily relied on cooperation with specific organizations and direct outreach in sex work settings, is limitation, as well as snowball sampling technique. While this approach enabled us to engage with certain groups of MFSWs, it likely excluded those who operate in more isolated or underground environments, resulting in a sample that may not fully represent the broader population. We also attempted online recruitment as part of our strategy. However, this approach did not yield the desired results. Feedback from participants indicated a lack of trust and engagement with digital platforms for recruitment, with some sex workers expressing concerns about privacy and safety when approached online. This highlights the challenges of reaching certain groups via online channels and suggests that this method may not be effective for this population. Given these limitations, future research should consider exploring alternative recruitment methods. This could include building trust over time with harder-to-reach groups, expanding collaborations with informal or community-based networks, and adopting a more personalized, face-to-face approach where possible. These strategies may offer more success in reaching a diverse and representative sample of MFSWs, especially those working in more hidden or marginalized settings. Due to the high heterogeneity of MFSW and the fact that healthcare situation in Berlin can differ from other cities of Germany, the findings of this study might not be generalizable to all MFSWs in Germany and also all sex workers, because the study focus was on migrant female sex workers.

Moreover, study’s sample size is small and lacks diversity, particularly in terms of participants from underrepresented regions such as Asia, Balkan and Baltic countries, Latin America, and Africa and women exposed to human trafficking and forced prostitution. Those who were not reached may also be confronted with other problems such as racism, which was not identified as a barrier in this study. The experiences of such a narrow sample may not fully represent the broader population of MFSWs and reduce the validity and generalizability of the findings.

One significant limitation of this study is the reliance on self-reported retrospective data collected through semi-structured interviews. This method is inherently vulnerable to recall bias, as participants may not accurately remember events or experiences, and social desirability bias, where participants may provide responses they believe are expected or more socially acceptable rather than their true experiences (49). These biases can potentially lead to inaccuracies in the data and affect the validity of the findings.

Although anonymity was maintained to encourage honest responses, it is important to acknowledge that these biases could not be entirely mitigated. Future studies may consider utilizing additional methods, such as triangulation with observational data or surveys, to validate self-reported data and further reduce the influence of such biases. These limitations should be considered when interpreting the study’s results as they may impact the overall generalizability and accuracy of the findings.

The study team consists of native speakers of different languages, such as Russian, Ukrainian, Bulgarian, German, Rumanian, Vietnamese, Turkish, but we still could not cover all languages due to heterogeneity of the group. All participants were fluent in English, and five interviews were conducted not in a mother tongue of participant, which can influence the quality and depth of the data collected, as participants might have struggled to fully express their experiences. To minimize the bias, we ensured that interviewers were fluent in the languages used and made extra efforts to clarify and confirm participants’ answers throughout the process. While professional translation services were not employed during the interviews, we carefully reviewed the transcripts for any ambiguities or misinterpretations.

## 5 Conclusion

The utilization of health services by migrant female sex workers is a complex issue involving a wide range of barriers at the system, provider, and patient levels. The qualitative approach of this study allowed to understand how these multilevel factors affect the health-seeking behaviors of MFSW and to reduce the knowledge gap of barriers to use health and mental health services by MFSW in Berlin. Moreover, firsthand information has been collected on needs for actions to make the use and access to health services easier for MFSW. This information could help policymakers, healthcare providers, and advocates for sex workers develop acceptable, affordable, and accessible health services. Future research with a more diverse and representative sample is needed to better capture the full range of experiences within this population and improve the health services utilization and health of MFSW, as part of the effort to protect the right of humans to health.

## Data Availability

The original contributions presented in the study are included in the article/supplementary material, further inquiries can be directed to the corresponding author.
